# The associations between caregivers’ psychological characteristics and patients’ apathy symptoms in multiple sclerosis

**DOI:** 10.1007/s10072-025-08463-6

**Published:** 2025-09-23

**Authors:** Maria Cropano, Giacomo Lus, Mariachiara Gaita, Lidia Ammendola, Daniela Malangone, Elisabetta Signoriello, Simona Raimo

**Affiliations:** 1 Department of Health Sciences, “Magna Graecia” University of Catanzaro, Catanzaro, Italy; 2 UOSD Second Neurology, Multiple Sclerosis Center, University of Campania “Luigi Vanvitelli”, Napoli, Italy; 3 Department of Medical and Surgical Sciences, University of Campania “Luigi Vanvitelli”, Napoli, Italy; 4 Department of Psychology, University of Campania “Luigi Vanvitelli”, Caserta, Italy; 5 Department of Medical and Surgical Sciences, “Magna Graecia” University of Catanzaro, Catanzaro, Italy

**Keywords:** Apathy, Multiple sclerosis, Caregiver burden, Coping strategies, Resilience

## Abstract

**Background/objectives:**

Apathy is a common neuropsychiatric symptom in people with Multiple Sclerosis (PwMS), and is significantly associated with increased dependency on caregivers, intensifying their emotional and physical burden. The present study aims to evaluate apathy from the perspectives of both PwMS and caregivers, considering the MS clinical phenotype; and to explore the impact of caregiver-reported patient apathy on their distress, particularly in relation to their coping strategies and resilience.

**Methods:**

Eighty dyads of PwMS and caregivers completed the self-evaluation or caregiver version of the Apathy-Motivation Index, which assesses apathy in terms of behavioral activation, social motivation, and emotional sensitivity. Caregivers also completed the Zarit Burden Interview and the Caregiver Burden Inventory to assess burden, the Coping Orientation to Problems Experienced to evaluate coping strategies, and the Connor-Davidson Resilience Scale to measure resilience.

**Results:**

Caregivers consistently reported higher levels of perceived patient apathy than PwMS, particularly in terms of behavioral apathy. Notably, higher levels of emotional apathy in PwMS, as perceived by caregivers, were strongly associated with increased psychological distress and burden. Furthermore, caregivers relying on maladaptive coping strategies experienced significantly greater levels of emotional exhaustion and role strain related to caregiver-reported patient apathy.

**Conclusions:**

These findings highlight the need to comprehensively assess all dimensions of apathy and integrate the caregiver’s perspective in its assessment. Tailored interventions should focus on reinforcing adaptive coping mechanisms to mitigate caregiver distress, associated with caregiver-reported patient apathy, ultimately improving the well-being of both PwMS and their caregivers.

## Introduction

Multiple Sclerosis (MS) is a chronic, progressive autoimmune disorder that primarily affects the central nervous system, leading to demyelination and subsequent neurodegeneration [1]. It is estimated that approximately 2.8 million individuals worldwide are affected by MS [2]. The disease manifests with a wide range of neurological symptoms, including muscle weakness, sensory disturbances, visual impairments, and coordination difficulties [3], as well as cognitive dysfunction [4] and neuropsychiatric symptoms [5, 6].

Among neuropsychiatric symptoms observed in MS, depression and anxiety have been extensively indagated [7] and are well-documented for their significant impact on both people with MS (PwMS) and their caregivers. These disorders frequently worsen the caregiving burden, leading to increased stress, emotional exhaustion, and poor physical health in caregivers [8, 9].

However, despite the high prevalence of apathy in PwMS, affecting approximately 40% of them [10, 11], it has received less attention in the context of caregiver burden. Apathy, encompassing cognitive, emotional, and behavioral dimensions, is characterized by a notable reduction in goal-directed behavior, motivation, and emotional responsiveness [12]. This condition not only negatively impacts PwMS but also significantly burdens caregivers. The lack of motivation, social withdrawal, and diminished engagement in daily activities associated with apathy [13] may lead to increased dependency on caregivers, exacerbating stress, emotional exhaustion, and overall caregiver burden [8], often culminating in burnout. The coping strategies caregivers employ to manage these challenges are critical in determining the extent of the burden experienced [14]. According to Lazarus and Folkman’s transactional model of stress and coping [15], individuals’ adaptation to stress is influenced by the coping strategies they employ. Positive reappraisal of the caregiving experience in the face of chronic stressors may enhance caregivers’ resilience and responsiveness to interventions.

Previous research on other neurodegenerative disorders, such as dementia and Amyotrophic Lateral Sclerosis, has demonstrated that apathy is associated with increased caregiver stress, diminished well-being, and a heightened caregiving burden [16, 17]. Similarly, Figved and colleagues [8] reported that apathy in PwMS was significantly correlated with greater caregiver distress and lower quality of life, even after adjusting for disability levels. These findings underscore the need for further research to elucidate how apathy manifests across different MS phenotypes and to develop targeted support strategies for both PwMS and their caregivers. However, in their study, Figved and colleagues [8] assessed apathy using the Neuropsychiatric Inventory (NPI; [18]), an informant-based interview that evaluates a range of neuropsychiatric symptoms, including apathy, but does not provide a comprehensive assessment of all apathy domains. Moreover, so far, no studies have examined the differences in self and caregiver-reported apathy considering separately the MS clinical phenotype. Indeed, MS phenotypes (i.e., relapsing–remitting MS, RRMS or progressive MS, PMS) have well-documented differences in cognitive [19, 20] and behavioral profiles [5]and to explore this difference could be essential to understand the mechanisms underlying apathy in MS better and to develop tailored interventions for PwMS and their caregivers.

Therefore, the present study aims to assess apathy from the dual perspectives of PwMS and their caregivers, with particular attention to the influence of MS clinical phenotype. Additionally, this study seeks to examine the associations between caregiver-reported patient apathy and caregiver burden, considering the role of caregivers’ psychological characteristics, specifically coping strategies and resilience. By adopting a comprehensive approach, this study aims to enhance the understanding of these complex dynamics and contribute to the development of tailored interventions for PwMS and their caregivers.

## Materials and methods

### Participants

A total of 80 PwMS (50 females, mean age 50.34 ± 13.53) and their caregivers, typically spouses or family members (39 females, mean age 48.85 ± 13.46) were enrolled at the Multiple Sclerosis Center of the Second Neurological Division at the University of Campania “Luigi Vanvitelli” in Napoli, Italy. PwMS were classified into two subgroups according to their clinical phenotype: 60 PwMS with RRMS (PwRRMS; mean age 47.82 ± 13.29) and 20 with a PMS (PwPMS), including both secondary and primary forms (mean age 57.90 ± 11.50). Inclusion criteria required: the absence of mild or major neurocognitive disorders (according to the Diagnostic and Statistical Manual of Mental Disorders, 5th edition, DSM-5; [21]), preserved general cognitive functioning (indicated by a Montreal Cognitive Assessment score within the normal range, MoCA; [22]), and a disability level not exceeding a score of 7 on the Expanded Disability Status Scale (EDSS; [23]). Additionally, both PwMS and their caregivers were excluded if they had a history of psychiatric disorders, head trauma, other neurological disorders, or substance abuse. The study adhered to the ethical principles of the Declaration of Helsinki and received approval from the local ethics committee. Written informed consent was obtained from all participants before enrollment.

### PwMS assessment

Demographic data (age, sex, level of education), medical history (age at disease onset, disease duration), level of disability (EDSS), and current pharmacological treatments were collected for all participants. Following this, participants underwent a neuropsychological assessment, which included:

- the MoCA ([22]), a brief screening tool designed to assess global cognitive functioning, evaluating memory, attention, executive functioning, visuospatial skills, language, and orientation. The total score ranges from 0 to 30, with a cut-off score of 15.5 used to exclude cognitive impairment in the Italian population.
- the self-report version of the Apathy Motivation Index (AMI-SE; [24]) was used to assess apathy. It consists of 18 items rated on a 5-point Likert scale, ranging from 0 (completely true) to 4 (completely untrue). It evaluates apathy across three dimensions: (1) Behavioral Activation (BA; items 5, 9, 10, 11, 12, 15), which refers to the tendency to self-initiate goal-oriented behavior, such as “I get things done when they need to be done, without requiring reminders from others”; (2) Social Motivation (SM; items 2, 3, 4, 8, 14, 17), which assesses engagement in social interactions, for example, “I start conversations without being prompted”; and (3) Emotional Sensitivity (ES; items 1, 6, 7, 13, 16, 18), which evaluates emotional responsiveness, such as “I feel awful if I say something insensitive.” All items are reverse-scored so that a higher score indicates more severe apathetic symptoms within each subscale. Scores are calculated as the mean rating for each subscale as well as for the total scale.
- the Beck Depression Inventory-Second Edition (BDI-II; [25]) to assess the severity of depressive symptoms (i.e. sadness, pessimism, loss of pleasure, guilty feelings, suicidal ideation or wishes, crying, agitation, loss of interest, loss of energy, concentration difficulty, tiredness or fatigue, loss of interest in sex). It includes 21 items, and participants are required to choose the point scale (from 0 to 3) that best describes how they felt in the last 2 weeks. The total score ranges from 0 to 63, with higher scores reflecting higher levels of depression.

### Caregiver assessment

Demographic data (age, sex, level of education) were collected for all participants. Following this, caregivers completed the following questionnaire:

- the caregiver version of AMI (AMI-CG; [24]) to assess caregivers’ perception of patients’ apathy. Caregivers will be asked to rate, on a five-point Likert scale, how accurately each statement describes the patient’s thoughts and behaviours. Scoring and interpretation are the same use in the self-report version.
- the Caregiver Burden Inventory (CBI; [26]), to evaluate caregiver burden. It includes 24 items on a 5-point Likert scale ranging from 0 (not at all descriptive) to 4 (very descriptive). The CBI explores five dimensions of stress: time-dependence (TD), developmental (D), physical (P), social (S), and emotional (E) burden. Specifically, the time-dependence burden reflects the time required for caregiving and its impact on the other caregiver’s activities; the developmental burden describes the caregiver’s feeling of being “off-time” in their own life’s progression; the physical burden describes the caregiver’s fatigue and physical health; the social burden reflects the role conflict and social isolation of caregiver; the emotional burden describes the emotional challenges and strain of providing care. The total score ranges from 0 to 96. Higher scores indicate a greater caregiver burden.
- the Zarit Burden Inventory (ZBI; [27]) is a self-administered questionnaire that explores various aspects related to the caregiver’s quality of life, psychological distress, guilt, financial difficulties, and social and family challenges resulting from their care-giving role. It includes 22-item score on a 5-point Likert scale ranging from 0 (never) to 4 (nearly always), with a range score from 0 to 88. Higher scores indicate greater caregiver burden: scores from 0 to 20 indicate little or no burden, from 21 to 40 mild to moderate burden, from 41 to 60 moderate to severe burden, and from 61 to 88 severe caregiver burden.
- the Coping Orientation to the Problems Experiences-new Italian version (COPE-NVI; [28]) describes the caregiver’s coping strategies that can be used to reduce or eliminate a stressor. It includes 60 items on a 4-point Likert scale, ranging from 1 (I usually don’t do this at all) to 4 (I usually do this a lot). COPE-NVI explores 13 coping strategies, that can be divided into five large and mostly independent sections: (a) social support, (b) avoidance strategies, (c) positive attitude (d) planning/activity and (e) turning to religion. The total score ranges from 60 to 240 with higher scores indicating greater psychological well-being in the face of stressful situations.
- the Connor-Davidson Resilience Scale (CD-RISC-25; [29]) reflects the caregiver’s ability to manage stress, cope with adverse experiences, and respond to stressful events. It consists of 25 items on a 5-point scale ranging from 0 (Not true at all) to 4 (I true nearly all the time) and the total score ranges from 0 to 100, with higher scores reflecting greater resilience. The total score can be interpreted as follows: low resilience (0–73), poor resilience (74–82), moderate resilience (83–90), and high resilience (91–100).

### Statistical analysis

The distribution of apathy scores (AMI total and subscale scores) was examined using the Kolmogorov–Smirnov test, which confirmed normality (D ≤ 0.099, *p* ≥ 0.050). Consequently, parametric statistical analyses were performed. Unpaired t-tests were performed to identify differences in demographic, clinical, and behavioral variables separately for PwMS and caregivers, stratified by clinical phenotype (i.e., RRMS, PMS). Additional paired t-tests were conducted to evaluate differences in apathy scores based on the perspective of evaluation (AMI-CG vs. AMI-SE), both for the entire PwMS group and within clinical phenotype subgroups.

A hierarchical multiple regression analysis was performed to investigate the relationship between caregiver-reported patient apathy (independent variable, step 1) and caregiver burden (dependent variable), adjusting for psychological factors (coping strategies and resilience; step 2). Seven separate models were analyzed, corresponding to different caregiver burden outcomes (total ZBI score, total CBI score, and CBI subscales). The strength of associations was assessed using beta coefficients and p-values, while R² values indicated the proportion of variance explained by the predictors. The significance level was set at an alpha level of < 0.05, and Bonferroni correction for multiple comparisons was applied. All analyses were performed using SPSS v. 23.0 (SPSS, Inc. Chicago, IL, United States).

## Results

### Comparison of demographic, clinical, and behavioral variables in PwMS and caregivers by clinical phenotype

Comparison results showed a significant difference between the two groups of PwMS. Specifically, PwPMS had older age (t = −3.031, *p* = 0.003) and higher levels of physical disability (EDSS; t = −8.679, *p* < 0.001) than PwRRMS (Table 1a). Regarding caregivers, significant differences emerged based on the clinical phenotype of the care recipient. Caregivers of PwPMS (CgPMS) reported significantly higher burden levels compared to caregivers of PwRRMS (CgRRMS), as evidenced by higher total scores on the ZBI (t = −3.486, *p* = 0.001), the CBI (t = −3.377, *p* = 0.002), and the CBI-TD subscale (t = −3.501, *p* = 0.001; Table 1b). No other significant differences were observed. A comprehensive summary of demographic, clinical, and neuropsychological data is provided in Table 1.

**Table 1:** Demographic, clinical, and behavioural characteristics of people with multiple sclerosis and their caregivers according to clinical phenotype

**a)**	**PwRRMS** (*n* = 60)	**PwPMS** (*n* = 20)	**PwMS** (*n* = 80)		
	Mean ± SD	Mean ± SD	Mean ± SD	t	*p*
Age	47.82 ± 13.29	57.90 ± 11.50	50.34 ± 13.53	−3.031	0.003**
Sex (F/M)	39/21	11/9	50/30	-	-
Education	13.18 ± 3.84	11.50 ± 4.57	12.76 ± 4.07	1.482	0.149
Age at onset	35.80 ± 12.63	43.55 ± 13.86	37.74 ± 13.29	−2.319	0.023*
Disease duration (months)	131.03 ± 97.10	166.75 ± 129.11	139.96 ± 106.27	−1.307	0.195
Disease duration (years)	10.95 ± 7.88	13.89 ± 10.76	11.68 ± 8.71	−1.124	0.271
EDSS	2.04 ± 2.14	5.44 ± 1.20	2.87 ± 2.44	−8.679	< 0.001**
MoCA	23.18 ± 3.07	20.45 ± 4.52	22.50 ± 3.66	2.515	0.019*
BDI-II	12.55 ± 12.66	12.75 ± 9.33	12.60 ± 11.86	−0.065	0.948
AMI-SE TOT	1.21 ± 0.51	1.25 ± 0.60	1.22 ± 0.53	−0.294	0.769
AMI-SE BA	0.99 ± 0.56	0.88 ± 0.59	0.96 ± 0.57	0.731	0.467
AMI-SE SM	1.39 ± 0.67	1.41 ± 0.99	1.40 ± 0.76	−0.093	0.927
AMI-SE ES	1.26 ± 0.67	1.47 ± 0.80	1.31 ± 0.70	−1.140	0.258
**b)**	**CgRRMS** (*n* = 60)	**CgPMS** (*n* = 20)	**CgMS** (*n* = 80)		
	Mean ± SD	Mean ± SD	Mean ± SD	t	*p*
Age	48.42 ± 13.94	50.15 ± 12.16	48.85 ± 13.46	−0.496	0.621
Sex (F/M)	29/31	10/10	39/41	-	-
Education	14.14 ± 3.79	12.85 ± 4.20	13.81 ± 3.91	1.273	0.207
AMI-CG TOT	1.40 ± 0.46	1.64 ± 0.70	1.46 ± 0.54	−1.740	0.086
AMI-CG BA	1.30 ± 0.49	1.41 ± 0.85	1.33 ± 0.60	−0.565	0.578
AMI-CG SM	1.51 ± 0.63	1.69 ± 0.66	1.55 ± 0.64	−1.073	0.287
AMI-CG ES	1.40 ± 0.58	1.83 ± 0.88	1.51 ± 0.68	−2.029	0.053
ZBI	15.15 ± 11.32	26.30 ± 15.22	17.94 ± 13.23	−3.486	0.001**
CBI TOT	6.55 ± 10.71	19.65 ± 16.20	9.82 ± 13.47	−3.377	0.002**
CBI-TD	5.95 ± 10.47	16.80 ± 15.83	8.66 ± 12.82	−3.501	0.001**
CBI-D	1.10 ± 2.32	4.25 ± 5.47	1.89 ± 3.62	−2.498	0.021*
CBI-P	1.42 ± 2.80	2.75 ± 3.43	1.75 ± 3.01	−1.572	0.127
CBI-S	1.08 ± 2.34	3.70 ± 4.36	1.74 ± 3.16	−2.560	0.018*
CBI-E	0.67 ± 1.48	2.30 ± 3.06	1.08 ± 2.09	−2.297	0.031*
COPE TOT	119.15 ± 17.35	127.50 ± 22.31	121.24 ± 18.91	−1.731	0.087
COPE-SS	21.13 ± 6.40	22.35 ± 6.74	21.44 ± 6.46	−0.726	0.470
COPE-AS	20.50 ± 4.35	22.95 ± 5.89	21.11 ± 4.86	−1.987	0.050
COPE-PA	29.42 ± 6.45	30.50 ± 6.77	29.69 ± 6.51	−0.642	0.523
COPE-P	25.28 ± 6.98	26.20 ± 6.14	25.51 ± 6.75	−0.523	0.602
COPE-TR	22.82 ± 4.82	25.50 ± 15.65	23.49 ± 8.81	−1.182	0.241
CD-RISC-25	52.93 ± 18.49	50.05 ± 19.72	52.21 ± 18.72	0.594	0.554

*, < 0.05

**, ≤ 0.004 after Bonferroni correction are reported in bold for PwMS; **, ≤ 0.002 after Bonferroni correction are reported in bold for caregivers

*PwMS* people with Multiple Sclerosis, *CgMS* caregivers of people with Multiple Sclerosis, *PwRRMS* people with a Relapsing–Remitting Multiple Sclerosis, *PwPMS* people with a Progressive Multiple Sclerosis, *CgRRMS* caregivers of people with a Relapsing–Remitting Multiple Sclerosis, *CgPMS* caregivers of people with a Progressive Multiple Sclerosis, *M* Male, *F* Female, *SD* Standard Deviation, *EDSS* Expanded Disability Status Scale, *MoCA* Montreal Cognitive Assessment, *BDI-II* Beck Depression Inventory-Second Edition, *TOT* Total score, *AMI-SE* self-report version of Apathy Motivation Index, *AMI-CG* caregiver version of Apathy Motivation Index, *AMI-BA* Apathy Motivation Index Behavioural Activation subscale, *AMI-SM* Apathy Motivation Index Social Motivation subscale, *AMI-ES* Apathy Motivation Index Emotional Sensitivity subscale, *ZBI* Zarit Burden Inventory, *CBI* Caregiver Burden Inventory, *CBI-TD* Caregiver Burden Inventory Time-Dependence subscale, *CBI-D* Caregiver Burden Inventory Developmental subscale, *CBI-P* Caregiver Burden Inventory Physical subscale, *CBI-S* Caregiver Burden Inventory Social subscale, *CBI-E* Caregiver Burden Inventory Emotional subscale, *COPE* Coping Orientation to the Problems Experiences, *COPE-SS* Coping Orientation to the Problems Experiences Social Support subscale, *COPE-AS* Coping Orientation to the Problems Experiences Avoidance Strategies subscale, *COPE-PA* Coping Orientation to the Problems Experiences Positive Attitude subscale, *COPE-P* Coping Orientation to the Problems Experiences Planning/activity subscale, *COPE-TR* Coping Orientation to the Problems Experiences Turning to Religion subscale, *CD-RISC-25* Connor-Davidson Resilience Scale

### Self- vs. Caregiver-Reported apathy in PwMS: comparisons for the entire sample and by clinical phenotype

When considering the entire PwMS cohort, significant differences emerged between self-reported and caregiver-reported apathy scores. Caregivers perceived higher levels of patient’s apathy than PwMS, as reflected in the AMI total score (t = −3.881, *p* < 0.001) and the BA subscale score (t = −4.592, *p* < 0.001 (Table 2a).

**Table 2:** Self and caregiver-reported apathy for the entire sample and according to clinical phenotype

**a) All participants**				
	**PwMS** (*n* = 80)	**Caregiver** (*n* = 80)		
	Mean ± SD	Mean ± SD	t	*p*
AMI-TOT	1.22 ± 0.53	1.46 ± 0.54	−3.881	< 0.001**
AMI-BA	0.96 ± 0.57	1.33 ± 0.60	−4.592	< 0.001**
AMI-SM	1.40 ± 0.76	1.55 ± 0.64	−1.898	0.061
AMI-ES	1.31 ± 0.70	1.51 ± 0.69	−2.344	0.022*
**b) RRMS**				
	**PwMS** (*n* = 60)	**Caregiver** (*n* = 60)		
	Mean ± SD	Mean ± SD	t	*p*
AMI-TOT	1.21 ± 0.51	1.40 ± 0.46	−2.783	0.007**
AMI-BA	0.99 ± 0.56	1.30 ± 0.49	−3.652	0.001**
AMI-SM	1.39 ± 0.67	1.51 ± 0.63	−1.326	0.190
AMI-ES	1.26 ± 0.67	1.40 ± 0.58	−1.502	0.139
**c) PMS**				
	**PwMS** (*n* = 20)	**Caregiver **(*n* = 20)		
	Mean ± SD	Mean ± SD	t	*p*
AMI-TOT	1.25 ± 0.60	1.64 ± 0.70	−2.859	0.010**
AMI-BA	0.88 ± 0.59	1.41 ± 0.85	−2.792	0.012**
AMI-SM	1.41 ± 0.99	1.69 ± 0.66	−1.389	0.181
AMI-ES	1.47 ± 0.80	1.83 ± 0.88	−2.001	0.060

*, < 0.05

**, ≤ 0.012 after Bonferroni correction are reported in bold

*PwMS* People with Multiple Sclerosis, *RRMS* Relapsing–Remitting Multiple Sclerosis, *PMS* Progressive Multiple Sclerosis, *SD* Standard Deviation, *AMI-TOT* Apathy Motivation Index Total score, *AMI-BA* Apathy Motivation Index Behavioural Activation subscale, *AMI-SM* Apathy Motivation Index Social Motivation subscale, *AMI-ES* Apathy Motivation Index Emotional Sensitivity subscale

Analyses stratified by clinical phenotype revealed that in PwRRMS, caregivers reported significantly higher levels of patient’s apathy than PwMS, both in the AMI total score (t = −2.783, *p* = 0.007) and the BA subscale score (t = −3.652, *p* = 0.001 (Table 2b). Similarly, in PwPMS, a significant discrepancy emerged in the AMI total score (t = −2.859, *p* = 0.010) and in the BA subscale score (t = −2.792, *p* = 0.012) (Table 2c). These findings indicate that caregivers generally perceive greater apathy in PwMS than is self-reported by the patients.

### Association between caregiver-Reported patient apathy and caregiver burden: hierarchical regression analysis

A two-step hierarchical multiple regression analysis was conducted to assess the relationship between caregiver-reported patient apathy and caregiver burden, while adjusting for psychological characteristics.

For the ZBI total score, caregiver-reported patient apathy in Step 1 accounted for a significant portion of variance in caregiver burden (F = 6.424, *p* = 0.001; R² = 20.2%). Notably, the AMI-CG ES subscale emerged as the only significant predictor (β = 0.319, t = 2.232, *p* = 0.029), indicating that higher levels of caregiver-reported patient emotional apathy were associated with greater caregiver burden. When psychological variables were added in Step 2, the explanatory power increased to 38.3% (F = 4.835, *p* < 0.001; R² =38.3%), with the AMI-CG ES subscale retaining significance (β = 0.320, t = 2.224, *p* = 0.029). Additionally, the COPE-NVI Avoidance Strategies subscale emerged as a significant predictor (β = 0.373, t = 3.305, *p* = 0.002), suggesting that higher levels of caregiver-reported patient emotional apathy, combined with greater reliance on avoidance coping strategies, contributed to increased caregiver burden.

Similar patterns were observed across different burden measures. For the CBI total score, caregiver-reported patient apathy alone accounted for a significant proportion of variance in caregiver burden at Step 1 (F = 4.058, *p* = 0.010, R² = 13.8%). When psychological variables were introduced in Step 2, the explained variance increased substantially (F = 5.112, *p* < 0.001, R² = 39.7%). The most robust predictors were the AMI-CG ES subscale (β = 0.301, t = 2.114, *p* = 0.038) and COPE-NVI Avoidance Strategies (β = 0.390, t = 3.495, *p* = 0.001), indicating that greater caregiver-reported patient emotional apathy and reliance on avoidance-based coping strategies were associated with heightened caregiver burden.

A similar pattern was observed for the CBI-TD subscale, where caregiver-reported patient apathy accounted for 12.7% of the variance in Step 1 (F = 3.697, *p* = 0.015, R² = 12.7%). After adjusting for coping strategies and resilience, the model’s explanatory power increased to 36.2% (F = 4.414, *p* < 0.001, R² = 36.2%), with both the AMI-CG ES subscale (β = 0.294, t = 2.011, *p* = 0.048) and COPE-NVI Avoidance Strategies (β = 0.386, t = 3.363, *p* = 0.001) remaining significant predictors.

For the CBI-D subscale, Step 1 revealed a significant effect of caregiver-reported patient apathy (F = 6.412, *p* = 0.001, R² = 20.2%), which persisted in Step 2, where the explained variance increased to 38.5% (F = 4.868, *p* < 0.001, R² = 38.5%). Significant predictors included the AMI-CG ES subscale (β = 0.323, t = 2.249, *p* = 0.028) and COPE-NVI Avoidance Strategies (β = 0.341, t = 3.028, *p* = 0.003), reinforcing the association between emotional apathy, maladaptive coping, and caregiver burden.

Conversely, for the CBI-P subscale, caregiver-reported patient apathy alone did not emerge as a significant predictor in Step 1 (F = 3.735, *p* = 0.051, R² = 12.8%). However, when psychological factors were included in Step 2, the explained variance increased markedly (F = 4.275, *p* < 0.001, R² = 35.5%), with COPE-NVI Avoidance Strategies being the only significant predictor (β = 0.367, t = 3.182, *p* = 0.002).

For the CBI-S subscale, caregiver-reported patient apathy did not significantly predict burden at Step 1 (F = 0.960, *p* = 0.416, R² = 3.7%). However, in Step 2, the inclusion of psychological variables significantly improved the model (F = 5.284, *p* < 0.001, R² = 40.5%), with COPE-NVI Avoidance Strategies emerging as the primary predictor (β = 0.543, t = 4.896, *p* < 0.001).

Similarly, for the CBI-E subscale, caregiver-reported patient apathy alone did not yield significant results in Step 1 (F = 2.596, *p* = 0.059, R² = 9.3%). However, following the inclusion of coping and resilience factors, the model improved substantially (F = 4.632, *p* < 0.001, R² = 37.3%), with both the AMI-CG ES subscale (β = 0.299, t = 2.061, *p* = 0.043) and COPE-NVI Avoidance Strategies (β = 0.375, t = 3.299, *p* = 0.002) emerging the primary predictors.

Taken together, these findings suggest that greater caregiver-reported patient emotional apathy and a predominant reliance on avoidance-based coping strategies are consistently associated with higher levels of caregiver burden across multiple dimensions. Notably, these effects were particularly pronounced for overall caregiver burden (ZBI and CBI total scores) and time-dependent burden (CBI-TD, CBI-D, CBI-E), underscoring the critical role of these psychological factors in shaping the caregiving experience.

## Discussion

This study aimed to investigate the differences in the perception of apathy between PwMS and their caregivers, and to assess whether the levels of caregiver-reported patient apathy were associated with caregiving burden, considering the role of coping strategies and caregiver resilience.

Our findings revealed that the presence of apathy is not significantly influenced by the clinical phenotype (RRMS or PMS) in either patients or caregivers, suggesting that apathy in PwMS can occur independently at any stage of the disease [6]. This highlights the importance of considering apathy as a core symptom of MS, one that transcends clinical phenotypes and underscores the need for a systematic assessment and management of this symptom throughout the disease course. Notably, our study was the first to identify a significant difference in caregiver burden between those caring for PwRRMS and those for PwPMS, suggesting that the progressive phenotype may place unique and greater demands on caregivers, further amplifying their burden. In particular, CgPMS reported higher levels of burden, especially in terms of time spent on caregiving and the impact on daily activities (CBI-TD subscale). Individuals with severe disability often require more support in daily activities, such as mobility and medication management, increasing their dependence on caregivers. Such demands may lead caregivers to abandon work or recreational activities [30, 31], further contributing to caregiving burden. Regarding the differences in apathy reported by patients and caregivers, we found that caregivers frequently reported higher levels of perceived patient’s apathy compared to PwMS. Specifically, caregivers reported higher levels of patient’s apathy in terms of behavioral apathy (AMI-BA subscale). This result is consistent with previous studies on neurological disorders such as Alzheimer’s disease, Parkinson’s disease, and limbic encephalitis [32, 33]. Behavioral apathy is characterized by a significant reduction in initiative, participation in daily activities, hobbies, and social interactions [34]. In this context, caregivers play a crucial role in mitigating the effects of apathy by providing external motivation and structured support to encourage engagement. The reduction in goal-directed activities observed in PwMS may include an anosognosic component in their behavioral profile, which should be clinically differentiated from other components of apathy. Regarding the associations between caregiver-reported patient apathy, caregiving burden, and psychological characteristics, our findings demonstrated that higher levels of caregiver-reported patient’s apathy, combined with a greater reliance on maladaptive coping strategies, were associated with an increase in caregiving burden. Specifically, higher levels of caregiver-reported patient emotional apathy, combined with greater use of avoidance coping strategies, were associated with increased distress related to time demands (CBI-TD subscale), a sense of inactivity in one’s life progress (CBI-D subscale), and greater emotional stress (CBI-E subscale).

In conclusion, this study emphasizes that apathy in PwMS and their caregivers is not significantly influenced by clinical phenotype (RRMS or PMS). However, caregivers consistently perceive higher levels of apathy in patients than PwMS particularly in terms of BA. Furthermore, emotional apathy and avoidance coping strategies are strongly associated with caregiving burden.

Our findings highlight the importance of including the caregiver perspective into the multidimensional assessment of apathy in PwMS, as their reports may provide crucial information that patient accounts fail to capture. Psychoeducational interventions for PwMS should aim to raise awareness and enhance the management of apathy symptoms. At the same time, interventions that promote more adaptive strategies, such as problem-focused or emotion-focused coping, could potentially reduce perceived burden and enhance caregivers’ emotional well-being. In particular, structured programs such as cognitive behavioural therapy and mindfulness-based interventions can support caregivers in becoming more aware of their emotional responses, reducing reliance on avoidance, and engaging in more constructive problem-solving and emotional regulation.

These interventions may help mitigate the impact of apathy on both the psychological well-being and quality of life of PwMS and their caregivers.

We acknowledge several limitations of our study. First, the sample size of PwPMS is relatively small, which may restrict the generalizability of our findings. Second, the reliance on self-reported measures from both PwMS and caregivers could introduce bias, such as social desirability or recall bias, potentially impacting the accuracy of results. Future research should aim to enhance the robustness and applicability of findings. Longitudinal studies with larger samples of PwPMS, combined with objective clinical measures and qualitative methodologies, could offer a more comprehensive understanding of the dynamics of apathy and its effects on both PwMS and their caregivers.

## Data Availability

The raw data supporting the conclusions of this article will be made available by the authors on request.
